# Education of the Public

**Published:** 1899-04

**Authors:** A. T. White

**Affiliations:** New Castle, Ind.


ARTICLE V.

### EDUCATION OF THE PUBLIC.

A. T. WHITE, D. D. S., NEW CASTLE, IND.

It would seem that nothing could be added to the subject of "Dental Education of the Laity," after the very able manner in which Drs. Stine and Henshaw have presented it in preceding numbers of the Journal. However, a few additional thoughts have presented themselves to me, and here they are:

I heartily agree with both in regard to the education of the teacher. Only a short time ago a teacher came to me with a woe begone face and the startling information that a piece of one of his lower front teeth had broken off. Investigation revealed the true condition—a great piece of calculi had become dislodged. "Didn,t know there was such a thing!"

This is only one of many instances.

This lamentable ignorance on the part of the teacher is only exceeded by that of the parents. The child's hands and face and clothing must be scrupulously clean, but the poor little mouth has never a scrubbing and never a thought. Had the mother who despaired of giving her son an education because she could not get him sterilized in time for school, given him a twenty-five cent tooth-brush and put him to work, the greatest danger would have been removed.

The great success which the study of scientific temperance has achieved, as taught to-day, instills into one the hope that something may yet be accomplished in the line of dental education.

It would at least set the teacher, too often derelict in caring for his teeth, to thinking, and might put him to work in cleaning up his oral cavity, that he might teach by example as well as by precept.

Now, with regard to my suggestion at the Muncie meeting as to the propriety of a practicing dentist lecturing in the public schools: I still think that a plain, practical talk once or twice a year in every school-room in the country would be of inestimable value in instilling into the minds of the pupils a desire to know more of, and care more for, their teeth.

The pupil is very likely to speak of anything unusual which happens at school, and in that way the parent would be set to thinking as well.

The thoughtful, conscientious dentist has done much in talking to the people with whom he comes in contact in his practice to lead them to give more attention to their children's teeth, but there is a great majority which can be reached only through the public schools.

A man of principle and tact need say noting in such a lecture to indicate that he is "looking for a job", or that should offend his most touchy brother practitioner. If he has sufficient ability to talk intelligently and entertainingly to a roomful of children, he deserves what little advertising such a talk would give him.

Finally, I should hail as a mighty stride in the line of progress a law which required that ever school board and every township trustee be required to have at least two talks by a competent dentist during each school year before every school in his jurisdiction.

